# Olefin selectivity of K-Mn promoters on CoFe-ZSM-5 based catalyst in CO_2_ hydrogenation

**DOI:** 10.3389/fchem.2025.1562436

**Published:** 2025-02-25

**Authors:** Paula Maseko, Mduduzi N. Cele, Masikana M. Mdleleni

**Affiliations:** ^1^ Department of Chemistry, North-West University, Mahikeng, South Africa; ^2^ South African Institute for Advanced Materials Chemistry, University of Western Cape, Cape Town, South Africa

**Keywords:** ZSM-5, carbon dioxide hydrogenation, ion exchange, promoters, selectivity, conversion

## Abstract

The conversion of carbon dioxide (CO_2_), a major greenhouse gas, into light olefins is crucial for mitigating environmental impacts and utilizing non-petroleum-based feedstocks. Thermo-catalytic CO_2_ transformation into valuable chemicals offers a promising solution to this challenge. This study investigates the effect of potassium (K) and manganese (Mn) promoters on CO_2_ conversion and C_2_H_4_ selectivity over CoFe-ZSM-5 zeolites. Structural characterization via FTIR, pyridine-FTIR, and PXRD confirmed the successful incorporation of K and Mn into CoFe-ZSM-5 at 80°C without significant structural changes to the zeolite framework. BET analysis revealed that metal incorporation did not substantially alter the surface area, while SEM and TEM analyses confirmed the preservation of ZSM-5 spherical morphology. Fixed-bed reactor experiments conducted at 350°C and 20 bar demonstrated that K and Mn synergistically enhanced CO_2_ conversion efficiency and selectivity toward C_2_H_4_. The K-Mn/4Fe4Co-ZSM-5 catalyst (modified with 4% Co and 4% Fe) exhibited the highest performance, achieving 97% olefin selectivity. Furthermore, Mn and K promoters reduce the CO selectivity on the Co-Fe-ZSM-5 catalyst. These findings underscore the critical role of K and Mn in facilitating efficient CO_2_ activation and directing the reaction pathway toward valuable olefin products.

## 1 Introduction

The extensive use of fossil fuels has led to significant carbon dioxide (CO_2_) emissions, contributing to pressing environmental issues such as global warming and ocean acidification (Abanades et al., 2017). South Africa, facing electricity shortages due to the depletion of coal reserves (Errero J.L 2023), illustrates the urgent need for alternative solutions. CO_2_ is a greenhouse gas and an abundant and cost-effective carbon source. Addressing the environmental impacts of CO_2_ emissions involves capturing (Sun et al., 2023; Zhou et al., 2024), utilizing (Luo et al., 2023), and converting CO_2_ (Ou et al., 2022; Tang et al., 2022; Luo et al., 2023). An effective strategy for mitigating CO_2_ emissions involves chemically transforming CO_2_ and green hydrogen into valuable products through thermo-, photo--, and electro-catalysis. This approach not only reduces CO_2_ emissions but also provides essential feed-stock for various industries (Saeidi et al., 2014; Díez-Ramírez et al., 2017; Edelmannová et al., 2018; Tang et al., 2022; Gao et al., 2023). However, the hydrogenation of CO_2_ to olefins presents challenges, including low selectivity, catalyst deactivation, and difficulties in controlling C-O bond activation and C-C bond formation (Weber et al., 2021; Jiang et al., 2023). To overcome these challenges, researchers are developing high-performance catalysts, particularly iron-based ones, by adjusting their morphology, composition, and synthesis methods (Cele, 2014; Liu et al., 2022). Advances include exploring the effects of promoters, supports, and bifunctional catalysts to enhance olefin yields and improve catalyst stability (Weber et al., 2021). Despite progress, managing C-O activation and C-C coupling in long-chain hydrocarbon production remains a significant hurdle (Wei et al., 2021).

Recent studies have focused on bifunctional catalysts for CO_2_ hydrogenation to olefins and aromatics, with promising results from Fe-based catalysts enhanced by elements like K, Mn, and Zn. Li et al. (2023) reported that FeMnKH-ZSM-5 achieved 70% selectivity for C_5_-C_11_ hydrocarbons and 17% for C_2_-C_4_ olefins at 320°C. García-Hurtado et al. (2020) found that the structure and size of zeolites influenced product selectivity, with MFI zeolites maximizing aromatic production and nanosized zeolites increasing light olefin selectivity. Liang et al. (2022) demonstrated that K-Zn-Fe/ZSM-5 catalysts achieved 45.2% aromatics selectivity with 42.6% CO_2_ conversion. Xu et al. (2020) reviewed the synergistic effects of Mn and Na additives on Fe catalysts, which improved olefin selectivity and space-time yields. These findings highlight the critical role of catalyst composition, zeolite structure, and promoter interactions in optimizing CO_2_ hydrogenation to produce valuable chemicals. Additionally, significant advancements have been made in the thermo-catalytic conversion of CO_2_ into light olefins, methanol, ethanol, and aromatics (Satthawong et al., 2015; Li et al., 2017; Liu et al., 2021; Ojelade and Zaman, 2021; Wang and Zhang, 2023).

The synthesis of light olefins from CO_2_ has gained considerable attention due to their importance as building blocks in the chemical industry (Li et al., 2017; Ronda-Lloret et al., 2019; Ojelade and Zaman, 2021; Muñoz and Weidema, 2024). Although traditional methods for olefin production, such as steam cracking and the methanol-to-olefins process, are well established (Al-Shafei et al., 2023; Wen et al., 2023; Muñoz and Weidema, 2024). Recent developments in multifunctional catalysts combining metal oxide nanoparticles with zeolites offer a promising pathway for CO_2_ hydrogenation, albeit with challenges like co-product selectivity and insufficient olefin yields (Jiang et al., 2023; Wang and Zhang, 2023). The CO_2_ Fischer−Tropsch (CO_2_-FT) synthesis presents a promising route for converting CO₂ into light olefins with a higher single-pass yield (Raje et al., 1997; Visconti et al., 2016). This process involves converting CO₂ into olefins via the reverse water gas shift reaction (RWGS Equation 1) and the Fischer–Tropsch synthesis (FTS Equation 2):

$${CO}_{2}+H_{2}\;⇌\text{CO}+H_{2}O$$ (1)

$$nCO+2nH_{2}\to \;C_{n}H_{2n}+nH_{2}O$$ (2)

Iron-based catalysts are preferred for CO_2_-Fischer-Tropsch (FT) synthesis due to their high activity in both the reverse water-gas shift (RWGS) and FT reactions (Guo et al., 2022; Fedorov et al., 2023; Yang et al., 2023). However, using iron alone does not always lead to a high selectivity for light olefins; modification of the catalyst’s chemical composition and structure is required. Typically, iron catalysts for CO₂ hydrogenation to light olefins are supported by oxides or carbon materials (Chen et al., 2023; Weber et al., 2023), with promoters often added to improve the yield of desired hydrocarbons by optimizing product distribution (Jiang et al., 2021; Chen et al., 2023; Weber et al., 2023). Common promoters for iron CO_2_-FT catalysts include potassium (K) (Qin et al., 2023), sodium (Na) (Ni et al., 2023), and manganese (Mn) (Singh et al., 2023). Incorporating cobalt into the iron catalyst can significantly alter surface properties, such as morphology, electronic structure, and chemical composition (Jiang et al., 2021). These modifications enhance CO₂ conversion efficiency and product selectivity by optimizing interactions between the catalyst and reactants. Cobalt can also influence reaction selectivity (Fan et al., 2021a), and adjusting the cobalt-to-iron ratio and other catalyst parameters allows for tailoring the selectivity towards specific hydrocarbons, such as long-chain alkanes, olefins, and oxygenates (Tsakoumis et al., 2010). In the RWGS reaction, adding cobalt can affect product distribution, favoring the formation of gases like methane or higher hydrocarbons (Díez-Ramírez et al., 2017; Fan et al., 2021b; Jiang et al., 2021).

Promoters in these catalysts function as either electronic or structural enhancers or both (Liu et al., 2021; Cele et al., 2019). Structural promoters help shape and stabilize the catalyst’s active phase, leading to a more uniform dispersion on the support, which increases conversion rates. Electronic promoters modify local electron density near the catalyst’s active site by donating or withdrawing electron density, influencing conversion levels and product selectivity (Ni et al., 2023; Sholeha et al., 2023). For instance, potassium acts as an electronic promoter in FT synthesis, enhancing CO_2_ conversion and reducing methane yield while increasing the alkene/alkane ratio and promoting longer-chain hydrocarbons (Liu et al., 2021; Wang et al., 2023). Manganese serves a dual role as both a structural and electronic promoter for iron catalysts. When added to an iron catalyst, manganese suppresses methane formation and enhances the alkene/alkane ratio in both FT and CO_2_ hydrogenation, functioning effectively as both a structural and electronic promoter (Kostyniuk et al., 2020; Jiang et al., 2021).

This study builds on current advancements by modifying a Fe-ZSM-5 catalyst with cobalt and incorporating potassium and manganese as promoters. The primary goal was to develop a more efficient catalyst for CO_2_ hydrogenation to olefins. The catalyst comprised 4% cobalt and 4% iron, with 0.5% promoter concentrations. The performance of the synthesized catalysts was evaluated in a fixed-bed reactor and characterized using various techniques, including PXRD, FTIR, Pyridine-FTIR, XRF, N_2_ adsorption, SEM, TEM, and EDS coupled with SEM.

## 2 Experimental

### 2.1 Materials

Zeolite Socony Mobil-5 with a *SiO*_2_/*Al*_2_ *O*_3_ ratio of 50, Iron (III) nitrate nonahydrate, Cobalt (II) nitrate hexahydrate, Manganese (II) nitrate hydrate and Potassium nitrate were all obtained from Sigma Aldrich. All the materials were used without further purification.

### 2.2 Ion exchange (IE) synthesis method

The Fe-ZSM-5 zeolites were synthesized by treating 1 g calcined ZSM-5 with Fe(NO_3_)_3_ salt in 20 mL deionized water, at 80°C for 3 h, facilitating the exchange of H⁺ ions with iron. The resulting material was then calcined at 550°C for 6 h. For the preparation of cobalt-modified Fe-ZSM-5 (Co-Fe-ZSM-5), Fe(NO_3_)_3_ was replaced with Co(NO_3_)_2_ powder. Manganese and potassium promoters were introduced using the same ion exchange technique with Mn(NO_3_)_2_ and KNO_3_, respectively.

### 2.3 Characterization

The chemical characteristics of surface functional groups in the zeolite catalysts were analyzed using FTIR spectra obtained across the vibrational range of 400 cm^−1^–4,000 cm^−1^ at room temperature. The measurements were taken using a Perkin Elmer Infrared spectroscope with a powder catalyst sample. The data were recorded and stored using Perkin Elmer software version 10.03.07. Pyridine-FTIR was done using the same instrument. Pyridine adsorption was performed at 200°C for 30 min, followed by evacuation at the same temperature for another 30 min to remove physisorbed pyridine.

Phase analysis was performed using a D8-Advance X-ray diffractometer from Bruker, operating in continuous θ-θ scan mode with Cu-Kα radiation. The sample was positioned on a glass slide at the correct height. Measurements covered a 2θ range from 0.5° to 130° with a step size of 0.034°. Diffraction data were collected using a Lyn-Eye position-sensitive detector.

The morphology of the ZSM-5 catalysts was examined using a Hitachi HF-2000 TEM operating at 200 kV and a JEOL 6400F SEM. EDS analysis was incorporated into the SEM for atomic-level composition analysis. The morphology of the ZSM-5 catalysts was examined using a Hitachi HF-2000 TEM operating at 200 kV and a JEOL 6400F SEM. EDS analysis was incorporated into the SEM for atomic-level composition analysis. The morphology of the ZSM-5 catalysts was examined using a Hitachi HF-2000 TEM operating at 200 kV and a JEOL 6400F SEM. EDS analysis was incorporated into the SEM for atomic-level composition analysis.

The BET surface area, pore volume, and pore sizes of ZSM-5 were determined using a Microtrac 3300 Tristar Surface Area and Porosity Analyzer. Each sample, containing approximately 0.3 g of the material, underwent a degassing process before analysis. This involved heating the sample in the BET apparatus while flowing nitrogen (N2) at 90°C for 1 hour, followed by further heating at 400°C for 4 hours. After preparation, the samples were loaded onto the analysis station, and the adsorbate was evaluated at a temperature of −195°C. The BET data was collected following an additional six-hour period.

XRF spectrometry was conducted using a PANalytical Axios Wavelength Dispersive spectrometer to determine major element compositions at the Central Analytical Facilities of Stellenbosch University in South Africa. This spectrometer, equipped with an Rh tube, utilized various analyzing crystals, including LIF200, LIF220, PE 002, Ge 111, and PX1. The instrument also featured a gas-flow proportional counter and a scintillation detector, employing a gas mixture of 90% Argon and 10% methane. Primary element analysis was carried out on fused glass disks utilizing a 2.4 kW Rhodium tube.

### 2.4 Catalyst testing

The zeolites were subjected to a compressive force of 30 bar, crushed, and then sieved to obtain particles sized between 2 and 3 mm. Exactly 1.000 g of the catalyst was loaded into a stainless-steel tube reactor, which was securely sealed at one end with quartz wool.

For catalyst preparation, an initial reduction step was conducted using a 10% H_2_/N_2_ gas mixture, flowing at 50 mL/min under atmospheric pressure in a tube furnace. This reduction process lasted for 4 h at 450°C. Following the reduction, the reactor was gradually cooled to 260°C. The CO_2_ hydrogenation process was then carried out by introducing a high-pressure CO_2_/H_2_ gas mixture in a 1:4 ratio at a flow rate of 20 mL/min. This process occurred at 350°C under 20 bar pressure.

A Perkin Elmer Clarus 400 GC equipped with a 30 m × 530 μm Suoelco Carboxen column and a thermal conductivity detector was utilized to analyze the CO_2_ feed and CO product. Ethene, methanol, methane and pentene were analysed using a Shimadzu GC-2025 (Kyoto, Japan) with a Petro-Elite column (50 m length, 200 μm diameter) and a flame ionization detector. The column temperature was initially maintained at 40°C for 5 min before being increased to 200°C at a rate of 3°C/min. All measurements were performed in triplicate.

After each performance test, the steel tube reactor was thoroughly cleaned. This involved multiple rinses with ethanol and deionized water to prevent cross-contamination. The reactor was then dried. Subsequently, the stainless-steel reactor, fitted with silica wool, underwent gas chromatography (GC) analysis after N_2_ was introduced to confirm the removal of ethanol and any residual contaminants. CO_2_ conversion was calculated using the formula:

$${CO}_{2}\;conversion\;\left(\%\right)=\frac{{CO}_{2in}-{CO}_{2out}}{{CO}_{2in}}\times 100$$

where CO_2in_ and CO_2out_ were obtained from the amount of CO_2_ at the inlet and outlet, respectively. The selectivity of the hydrocarbon products was calculated using the following equation:

$$C_{n}H_{m}\;selectivity\;\left(\%\right)=\frac{n\times C_{n}H_{m}}{{CO}_{2\;in}-{CO}_{2\;out}-{CO}_{\;out}}\times 100$$

where C_n_H_m_ represents the individual hydrocarbon product amounts in moles. The selectivity of CO was calculated according to the following equation:

$$CO\;selectivity\;\left(\%\right)=\frac{{CO}_{OUT}}{{CO}_{2\;in}-{CO}_{2\;out}}\times 100$$

where CO_out_ represents CO at the outlet amount.

## 3 Results and discussion

### 3.1 Structural and textural properties of catalysts

**FIGURE 1:**
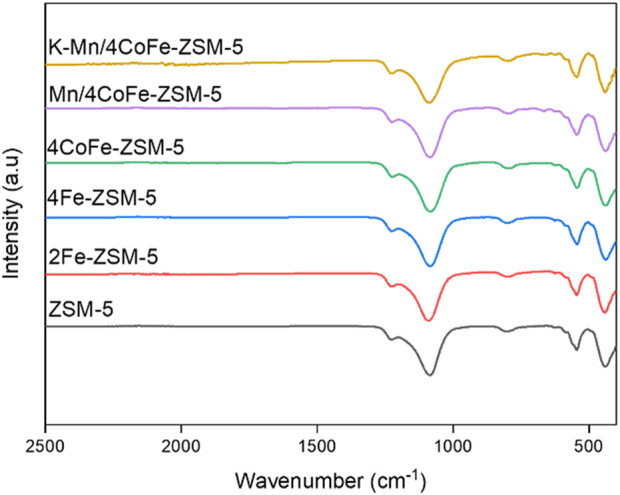
FTIR Plots of ZSM-5 and modified ZSM-5 zeolites.

Figure 1 presents the FTIR spectra of the calcined catalysts in the 3900–400 cm^−1^ range. Distinct absorption bands were observed at 1,087 cm^−1^, 795 cm^−1^, 547 cm^−1^, and 441 cm^−1^, corresponding to the asymmetric stretching vibrations characteristic of highly siliceous materials. The band at 441 cm^−1^ is attributed to T-O-T (T = metal) bending vibrations, while the 547 cm^−1^ band is linked to structure-sensitive vibrations from the double five-membered rings in the external linkage. During the ion exchange method, metal species occupy extra-framework positions within the ZSM-5 framework, which has a negative net charge. This occupation balances the zeolite charge, resulting in a stable interaction between the metal species and the zeolite structure. This interaction enhances the stability of the metal species and reduces their detachment and conglomeration. Additionally, absorption bands at 1,087, 795, and 441 cm^−1^ were associated with the symmetrical stretching of Si-O, asymmetrical stretching of Si-O, and the bending of Si-O/Al-O (SiO_4_ and AlO_4_ tetrahedron units), respectively. All samples displayed the primary characteristic vibrations of SiO_4_ and AlO_4_, indicating that the zeolite structure remained intact after modification.

**FIGURE 2:**
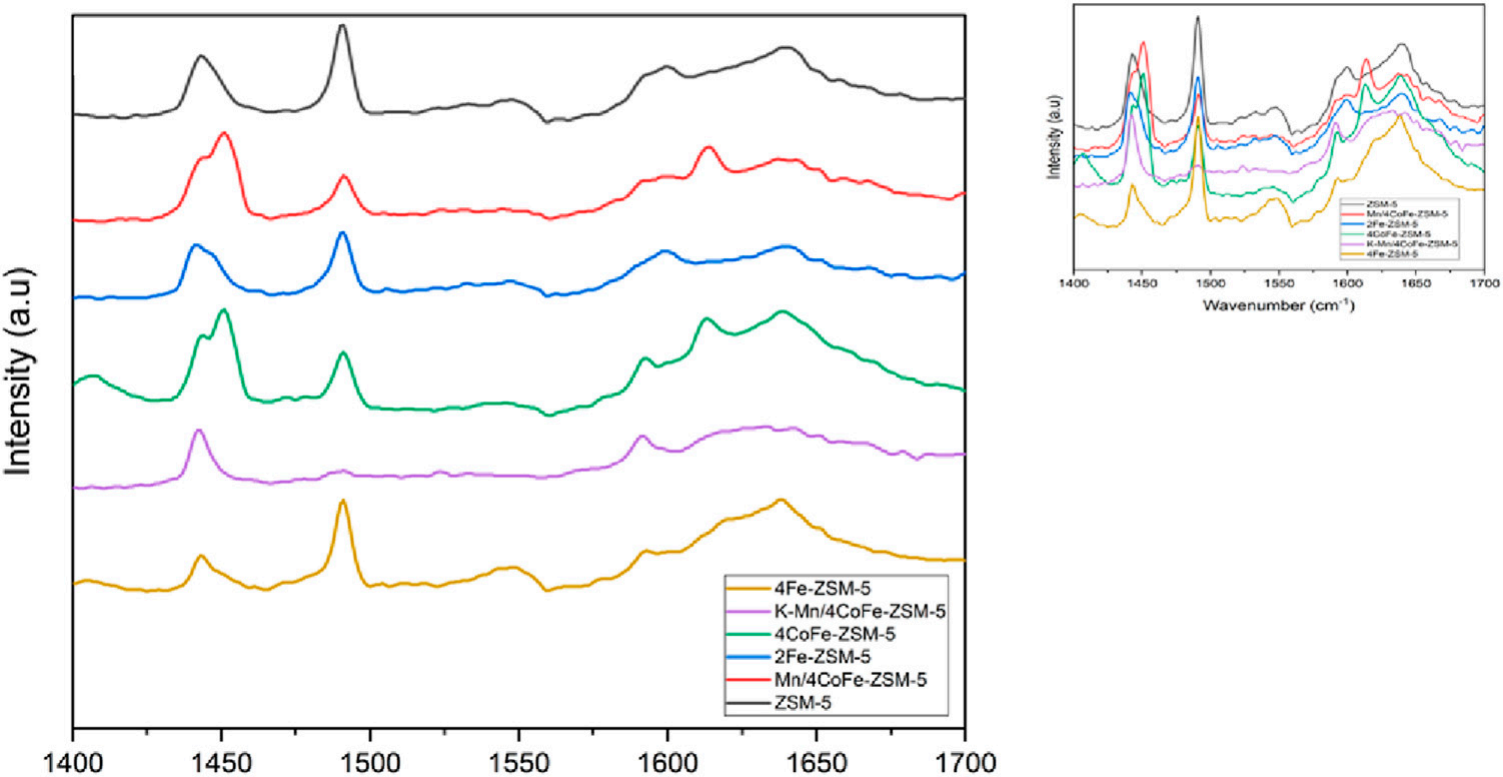
Pyridine DRIFTS adsorption spectra of ZSM-5, Fe-ZSM-5, CoFe-ZSM-5 catalysts.

The acidity profiles of ZSM-5 and modified ZSM-5 catalysts were examined, and the resulting adsorption spectra are shown in Figure 2. As reported by Kostyniuk et al. (2020), bands corresponding to Bronsted acid sites (BAS) were identified at 1,545 cm^−1^, while Lewis acid sites (LAS) were indicated at 1,445 cm^−1^. The band at 1,489 cm^−1^ was attributed to pyridine adsorbed on both BAS and LAS, as well as H-bonded pyridine. Upon incorporating Fe species into ZSM-5 (2Fe-ZSM-5 and 4Fe-ZSM-5), the catalysts displayed a band at 1,445 cm^−1^ associated with Lewis-bonded pyridine. The presence of pyridine adsorption via hydrogen bonding at 1,489 cm^−1^ confirmed the existence of weak and medium Bronsted acid sites. Additionally, a band at 1,545 cm^−1^, linked to BAS, was also detected.

For the adsorption spectra of 4CoFe-ZSM-5. The catalyst displayed a band at 1,445 cm^−1^, corresponding to Lewis-bonded pyridine. In this Lewis region (1,445 cm^−1^), a shift and peak splitting were observed compared to ZSM-5, consistent with observations by Dokania et al. (2020). This suggests the presence of multiple species with Lewis acidic character within the zeolite. A notable change of Bronsted acid sites (BAS) at 1,545 cm^−1^ was also observed. Peak splitting is observed with the introduction of Mn, indicated by a band at 1,624 cm^−1^ in the Mn/4CoFe-ZSM-5 catalyst, which was attributed to Lewis-bonded pyridine.

For K-Mn/4CoFe-ZSM-5, the Lewis acid sites (LAS) and Bronsted acid sites (BAS) were similar to those in the other catalysts, but they lacked the band at 1,489 cm^−1^, indicating a low presence of weak BAS. Due to the unique nature of the Lewis sites resulting from metal modification and the limited data on extinction coefficients, quantification of the acid sites could not be performed.

As shown in Figure 2, adding metals to ZSM-5 resulted in a significant change in Bronsted acid sites. These results suggest that metal incorporation into the ZSM-5 extra framework influences the Lewis acid sites while the Bronsted acid sites are affected. The reduction in Brønsted acidity hinders hydrogen transfer reactions, leading to increased production of light olefins and reduced production of longer-chain hydrocarbons.

**FIGURE 3:**
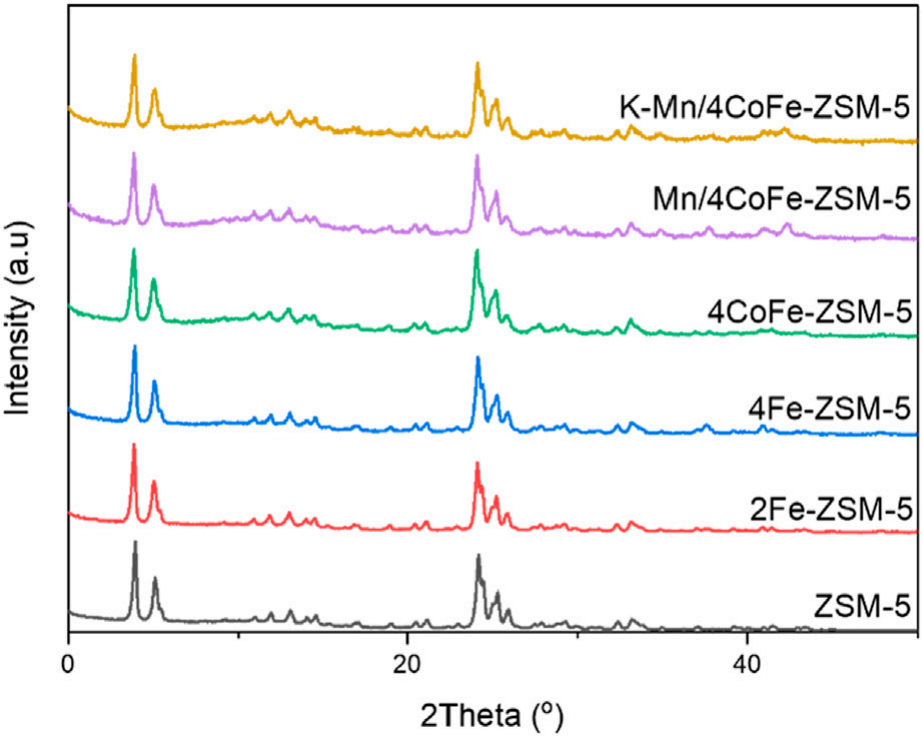
XRD patterns of ZSM-5 and modified ZSM-5 catalysts.

X-ray diffraction analysis was utilized to validate the crystallinity of the material. Figure 3 illustrates the XRD peaks for ZSM-5 and the modified ZSM-5 catalysts. All samples exhibited sharp and intense diffraction peaks, indicative of high crystallinity. No crystallinity alteration was observed when metals were loaded onto the zeolite. All ZSM-5-based adsorbents displayed peaks at 2*θ* = 7.9°, 8.9°, 23.0°, and 23.9°, corresponding to the (101), (020), (332), and (303) planes of the MFI structure of zeolite (JCPDS). The diffraction peaks of all modified ZSM-5 catalysts remained consistent with those of the parent ZSM-5 in the 7°–65° range, indicating retention of the ZSM-5 structure after metal treatment.

**FIGURE 4:**
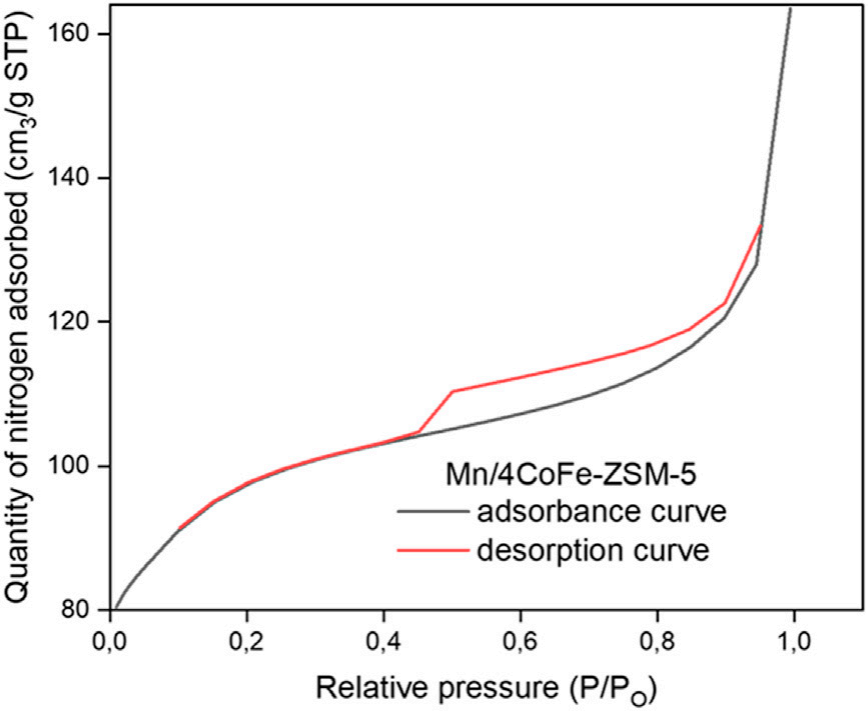
N_2_ adsorption/desorption isotherm curve of Mn/2Co-Fe-ZSM-5.

**FIGURE 5:**
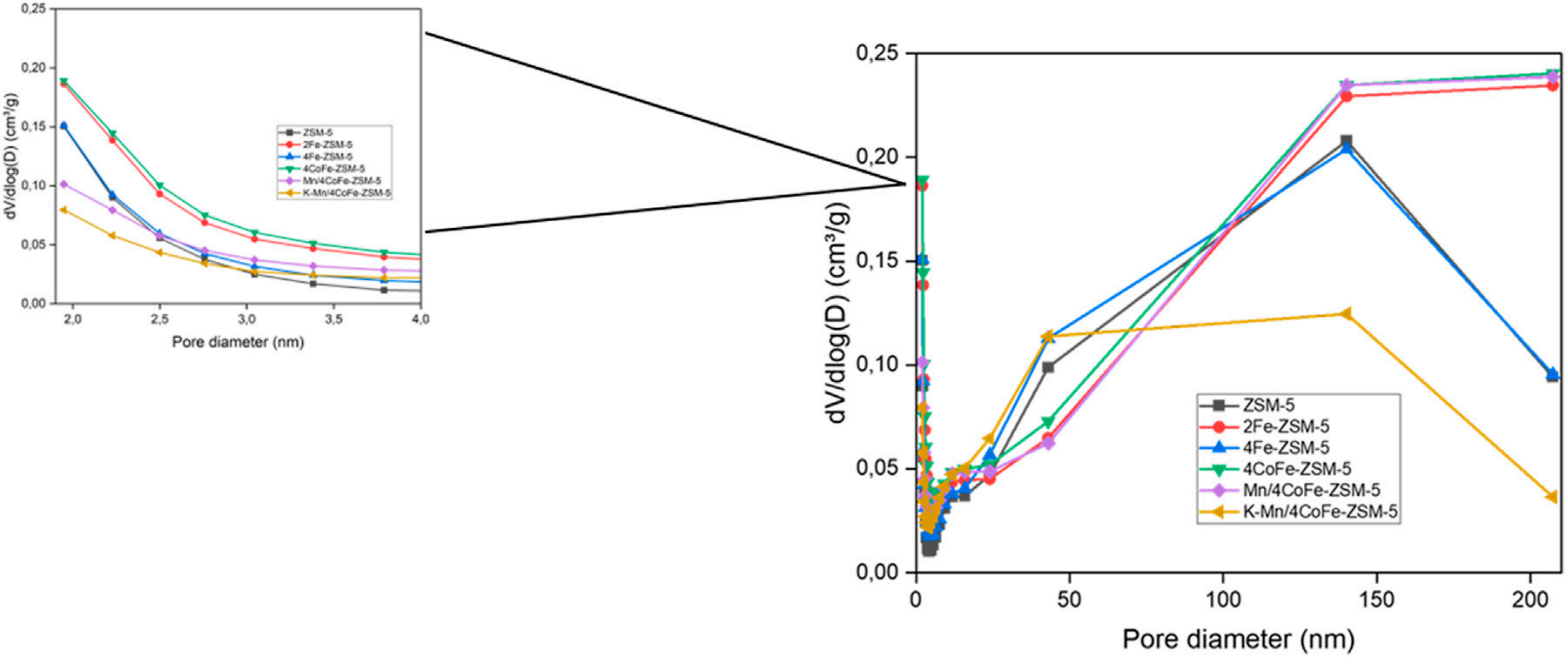
Pore size distribution of ZSM-5 and modified ZSM-5 catalysts.

Figure 4 illustrates the N_2_ adsorption-desorption isotherm curve of Mn/4CoFe-ZSM-5. Additional details can be found in the Supplementary Material. All catalysts exhibit type IV isotherms, which are characteristic of porous adsorbents. H4 hysteresis loops are observed at high relative pressures, indicating the presence of hollow spheres consisting of ordered mesoporous silica walls. The trend suggests increased N_2_ adsorption as pores become filled at high relative pressures (0.45 < *p*/*p*0 < 0.95). The absorption of nitrogen at pressure ratios of *P*/*P*0 = 0.3 and 0.9 is attributed to the presence of intra- and interparticle pores, respectively. Notably, as the amount of metal increased, the number of intraparticles changed while interparticle pores increased. These modifications led to alterations in the surface area, as observed in Table 1. The pore size distribution of the zeolites was determined using the Barrett Joyner Halenda (BJH) approach, as shown in Figure 5. The catalysts exhibited a concentrated pore size of 2.2 nm.

**TABLE 1:** Textural properties of modified ZSM-5 catalysts.

Catalyst	*SBET* ^a^ (*m* ^2^/*g*)	*Smicro* ^b^ (*m* ^2^/*g*)	*Vmeso* ^c^ (*cm* ^3^/*g*)	*Vmicro* ^b^ (*cm* ^3^/*g*)
K-Mn/4Co-Fe-ZSM-5	343	298	0.87	0.12
Mn/4Co-Fe-ZSM-5	358	296	0.87	0.12
4CoFe-ZSM-5	412	305	0.87	0.12
4Fe-ZSM-5	394	341	0.85	0.14
2Fe-ZSM-5	407	302	0.86	0.12
ZSM-5	419	372	0.83	0.15

Mesopore surface area and volume were obtained by subtracting micropore area from the total.

^a^ BET method.

^b^ t-plot method.

^c^ Vmeso = Vads ad p/p0 = 0.99−Vmicro.

The BET surface area, external surface area (Smicro), micropore volume, and mesopore volume of ZSM-5 catalysts obtained from the N_2_ adsorption/desorption isotherms are presented in Table 2. The introduction of Co, Fe, and metal promoters (Mn and K) into the structure of the ZSM-5. The micropore volume experiences a minor change as the metal loading increases, possibly due to metal deposition either on the surface or inside the micropores, indicating minimal volume change.

**TABLE 2:** Elemental content from XRF analysis.

Catalyst	Al content (%)	Si content (%)	Fe content (%)	Co-content (%)	Mn content (%)	K Content (%)
K-Mn/4CoFe-ZSM-5	1.2	34.2	4.4	5.3	0.6	1.5
Mn/4CoFe-ZSM-5	1.2	34.8	4.7	7.7	0.6	0.0
4CoFe-ZSM-5	1.3	38.5	2.1	2.1	0.0	0.0
4Fe-ZSM-5	1.3	38.7	5.1	0.0	0.0	0.0
2Fe-ZSM-5	1.4	40.6	2.3	0.0	0.0	0.0
ZSM-5	1.4	41.7	0.0	0.0	0.0	0.0

The ZSM-5 support displayed a BET surface area of 419 m^2^/g, which changes slightly with each metal loading but within the reported surface area range of ZSM-5. The combination of iron (Fe) and cobalt (Co) appears to promote improved dispersion of the metals as the metal loadings increase. An increase in the amount of metals on the catalysts might lead to a change in the surface area due to the dispersion of metals on the catalyst surface, resulting in a more significant number of active sites accessible to the reactant’s molecules for the reaction. The specific surface area of 4Fe-ZSM-5 is 394 m^2^/g, as determined by the BET method. After modifying the catalyst with 4% cobalt, the BET surface area slightly changed to 412 m^2^/g.

### 3.2 Morphology and elemental characterization

**FIGURE 6:**
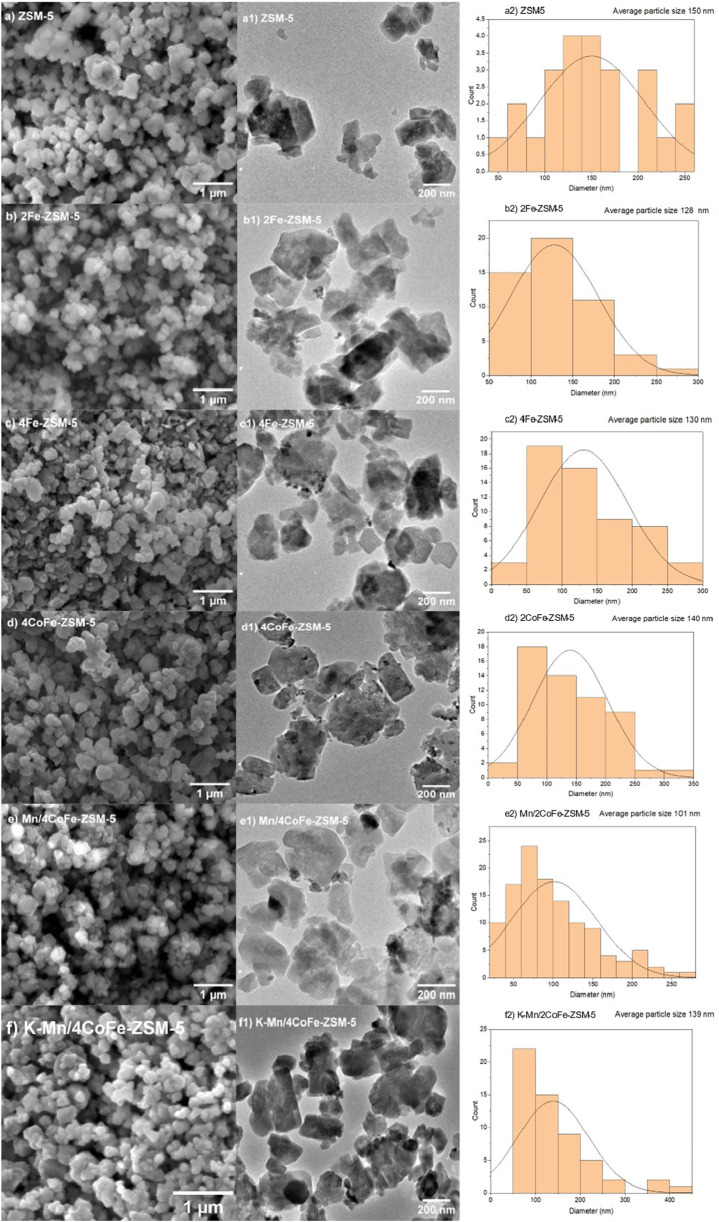
**(A–F)** SEM and TEM images of ZSM-5 and the modified catalysts, **(A1–F1)** histograms of particle size distributions of ZSM-5 and the modified catalysts **(A2–F2)**.

**FIGURE 7:**
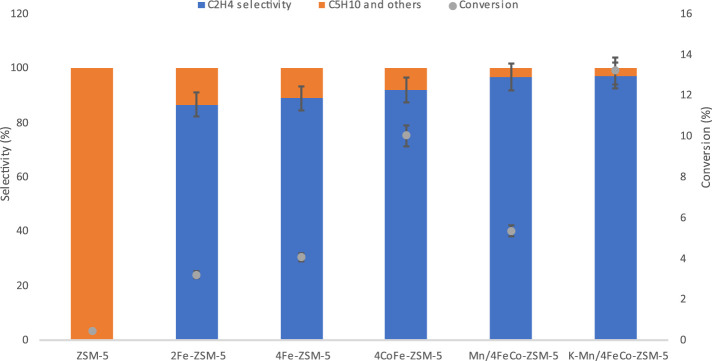
Hydrocarbon selectivity over ZSM-5 and modified ZSM-5 catalysts and CO_2_ conversion at 350°C and a pressure of 20 bar after a 2-hour duration.

Figures 6A–E showcases scanning electron microscope (SEM) images of both untreated ZSM-5 catalysts and those treated with metals. In the SEM micrographs of ZSM-5 zeolites (Figure 7A), the morphology of cuboids composed of aggregated crystal structures is observable. Notably, the absence of discernible textural alterations post-modification suggests that the ZSM-5 framework remained unchanged following the introduction of metals (Figures 7B–E). However, the morphology could benefit from improved resolution, predominantly consisting of condensed and clustered granular particles with a cubic and quasi-hexagonal form.

The particle size distribution was determined using the ImageJ program, revealing that all catalysts possess a mean particle size ranging from 101 to 150 nm, as indicated by the histograms in Figures 6A2–F2. Interestingly, the particle size distribution of the modified catalysts closely resembles that of the unmodified ZSM-5 catalysts after the introduction of metals. This particle size distribution across the modified catalysts indicates that differences in catalytic performance are likely influenced more by the nature of the promoters and their interactions with the CoFe-ZSM-5 framework.

Furthermore, a comprehensive elemental mapping study was conducted on the surface of ZSM-5 to unveil the distribution of Fe, Co, and Mn species (Supplementary Figures S12–S20). These images depict the spatial distribution of metals on a macroscopic level. Additionally, the TEM micrographs in Figures 6B1–F1 provide enhanced insight into the textural alterations. Ultimately, the remaining catalysts exhibited comparable properties, with relevant data accessible in the Supplementary Material.

Table 2 displays the quantities of aluminum (Al), silicon (Si), and other metals present in the modified ZSM-5. The table demonstrates the successful incorporation of metals into the ZSM-5 extra-framework. The XRF measurements reveal that more than 4% of Fe was incorporated into the zeolite 4Fe-ZSM-5. In addition, there was no noticeable decrease in the weight percentage of Fe for the Mn/4CoFe-ZSM-5 catalyst following modification. This was accomplished because of metal ions being retained in the extra-framework when potassium is exchanged onto the zeolite extra-framework. A similar phenomenon is seen on K-Mn/4CoFe-ZSM-5. The K was accurately substituted into the catalyst, whereas the amount of Mn was below the anticipated level. The aluminium (Al) content remained consistent, whereas the silicon (Si) content declined. Among the samples, K-Mn/4CoFe-ZSM-5 had the lowest Si content. The decrease in silicon content enhances the acidity of the catalyst, resulting in a greater preference for olefins (Jiang et al., 2020).

### 3.3 Catalytic performance

**FIGURE 8:**
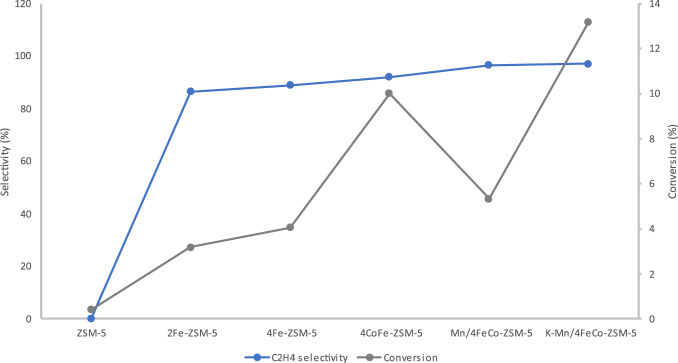
C_2_H_4_ selectivity over ZSM-5 and modified ZSM-5 catalysts and CO_2_ conversion at 350°C and a pressure of 20 bar after a 2-hour duration.

The catalytic activity experiments were conducted under a CO_2_ and H_2_ mixture at a temperature of 350°C and a pressure of 20 bar, following a reduction step at 450°C. A concise summary of the collected data after a 2-h duration during the reaction is provided in Table 3 and Figures 7, 8. According to Table 3, a CO_2_ conversion rate of 3.2% was observed with a 2 wt% Fe loading on the ZSM-5 support. The low CO_2_ conversion over metal-modified ZSM-5 is primarily due to the low weight percentage of the active metals. The selectivity of C_2_H_4_ (ethene) was measured to be 86.7%. Additionally, byproducts such as C_5_H_10_, CO, CH_4_, and CH_3_OH were also generated. Figure 7 and Table 3 illustrate that the catalyst with a deposition of 4 wt% Fe on ZSM-5 exhibited higher CO_2_ conversion to ethene compared to the catalyst with 2%wt Fe. Specifically, the 4Fe ZSM-5 catalyst achieved a CO_2_ conversion rate of 4.1% with a selectivity of 88.9% towards C_2_H_4_. Furthermore, the selectivity towards ethene was enhanced with higher weight percentages of Fe. It can be inferred that the conversion of CO_2_ increased as the quantity of deposited Fe on the support increased. The reduction in surface area with increasing Fe content did not adversely impact ethene selectivity, suggesting that selectivity is determined more by the characteristics of the active sites than by the surface area. These results are consistent with previous studies where the increase in metal loading also resulted in higher CO_2_ conversion and selectivity towards ethene, indicating that our proposed catalyst is competitive with those previously reported (Kostyniuk et al., 2020; Yuan et al., 2023a).

**TABLE 3:** Catalytic performance of ZSM-5 and modified ZSM-5 catalysts.

Catalyst	CO_2_ conversion percentage (%)	Product selectivity (%)		Hydrocarbon distribution (%)			
		CO	HC	CH_3_OH	CH_4_	C_2_H_4_	C_5_H_10_
K-Mn/4Co-Fe-ZSM-5	13.2	1.5	98.5	0.2	0.1	97.3	0.9
Mn/4Co-Fe-ZSM-5	5.3	2.4	97.6	0.6	0.1	96.7	0.2
4Co-Fe-ZSM-5	10.1	7.5	92.4	0	0.4	92.1	0
4Fe-ZSM-5	4.1	0	100	9.4	1.5	88.9	0.2
2Fe-ZSM-5	3.2	0	100	11.5	1.7	86.7	0.1
ZSM-5	0.4	0	100	0	100	0	0

Reaction at 350°C, under 20 bar pressure with CO_2_/H_2_ gas mixture, in a 1:4 ratio, at a flow rate of 20 mL/min at duration of 2 h.

The enhanced hydrocarbon selectivity was attributed to the effective conversion of CO_2_ into hydrocarbons, indicating that the active sites for Fischer-Tropsch synthesis of the Fe-ZSM-5 catalysts exhibited high activity. The occurrence of the Reverse Water Gas Shift (RWGS) reaction was attributed to magnetite iron (Fe_3_O_4_) or amorphous oxide phase. In contrast, chain growth necessitated the presence of a carbide phase, primarily χ-Fe_5_C_2_(Jiang et al., 2020). These findings align with reference (Dokania et al., 2020). The Fe-ZSM-5 catalyst primarily comprised the Fe_3_O_4_ phase, with small amounts of iron carbide, resulting in a reduced ability to produce long-chain hydrocarbons (C_5_H_10_). The Fe-ZSM-5 catalysts showed no selectivity to CO product.

Table 3 demonstrates a significant improvement in C_2_H_4_ selectivity and CO_2_ conversion upon deposition of Co onto the Fe-ZSM-5 catalysts. The selectivity 4CoFe-ZSM-5 exhibited a selectivity of 92.1% to C_2_H_4_ with a CO_2_ conversion rate of 10.1%. The notable improvement in CO₂ conversion and C_2_H_4_ selectivity arises from the 1:1 Fe to Co weight percentage ratio (Table 2), which is crucial for maximizing CO_2_ conversion efficiency and light olefin selectivity (Yang et al., 2017; Hua et al., 2023). This is attributed to the complementary catalytic functions and synergistic interaction between iron and cobalt. An enhanced CO selectivity is recorded, indicating the heightened reactivities of the RWGS reaction because of the presence of cobalt. Fe−Co contact during the hydrogenation reaction over the CoFe-ZSM-5 catalysts facilitated the creation of Co−Fe carbide, Co_2_C, and θ-Fe_3_C phases. This mechanism prevented the excessive hydrogenation of both CO and CH_3_OH intermediates into methane and light olefins into alkanes. The presence of both metallic and acidic functions on metal carbides is advantageous for the process of CO_2_ hydrogenation due to the easy absorption of CO_2_ in carbides. The addition of Co showed 7.5% selectivity to CO product.

An improvement in C_2_H_4_ selectivity of 96.7% was seen when the catalysts were modified with Mn. A decline in CH_4_ selectivity (0.08%) and CO_2_ conversion (5.3%) is observed. The decrease in hydrocarbon (HC) selectivity occurred because the addition of manganese (Mn) to iron (Fe) catalysts reduces the formation of methane (CH_4_) while enhancing the production of olefins. The addition of trace amounts of Mn can greatly enhance the activity of the catalyst (Chen et al., 2024). XRF analysis, as shown in Table 2, verified that the Mn/4Co-Fe-ZSM-5 catalyst contained 0.6 weight percent of manganese. Additionally, the decrease in CO_2_ conversion is accompanied by the increase in selectivity to C_2_H_4_. It is observed that the addition of Mn to 4CoFeZSM-5 reduced the CO selectivity from 7.5% to 2.4%.

Once again the surface area (Table 1) did not adversely impact the selectivity as the addition of K to the catalyst led to a substantial increase in CO_2_ conversion while the selectivity to C_2_H_4_ was still significantly high, even though the surface area decreased. The rise is attributed to the promotion of CO_2_ conversion facilitated by the addition of alkaline substances. In the Fischer-Tropsch process, excessive K deactivates the iron catalyst, making it essential to use only minimal amounts (Dorner et al., 2010). As confirmed by XRF small amounts of K were exchanged onto the catalyst.The addition of K to the catalyst considerably enhanced the selectivity of C_2_H_4_. The improvement in selectivity was a result of the promotion of K, which caused a notable shift in the production of olefins. The exceptional catalytic performance (13.2% CO_2_ conversion, 97.3% C_2_H_4_ selectivity) seen in the presence of potassium (K) can be attributed to the enhanced ability of CO_2_ chemisorption and the inhibition of H_2_ adsorption. The potassium also assisted in reducing the CO selectivity further as 1.5% selectivity to CO was obtained. This, in turn, improves the likelihood of C-C bond formation. Ethene (C_2_H_4_) was the predominant product obtained with all of the catalysts. This could be attributed to the limited re-adsorption of C_2_ products on the catalyst surface, which hindered the formation of C_3_-C_5_ products by chain growth. The rate of CO_2_ hydrogenation in unmodified ZSM-5 zeolite compared to metal-modified ZSM-5 zeolite can differ significantly due to the influence of metal species on the catalytic activity and reaction pathways. In its unmodified form, ZSM-5 zeolite exhibits very small intrinsic catalytic activity towards CO_2_ hydrogenation, primarily driven by its acidic sites and pore structure. However, the catalytic activity is limited compared to metal-modified counterparts (Yuan et al., 2023b). This is shown on Table 3. The activity evaluation was performed with triplicate measurements, yielding 97.3% ± 0.9% at a 95% confidence level. This highlights the precision of the measurements and the reproducibility of the results.

The increased selectivity to pentene, can be attributed to the catalytic properties of the K modified catalyst, which favors the production of long chain olefins. The incorporation of metals such as Fe, Co, K, and Mn likely alters the acid site distribution and balances hydrogenation and dehydrogenation steps, suppressing the formation of other hydrocarbons while enhancing pentene selectivity (Wang et al., 2024).

Metal modification significantly alters the activity of the CO_2_ hydrogenation reaction. The presence of metal species can enhance the adsorption of reactants and intermediates, thereby increasing the likelihood of reaction occurrence and improving the reaction rate at the reaction conditions. Different metals exhibited varying selectivities towards specific products, such as methanol, ethene and pentene (Table 3). Faster reaction kinetics would be expected over the metal modified ZSM-5 due to enhanced catalytic activity and increased availability of active sites provided by the metal species. Metal-modified ZSM-5 may require regeneration steps to maintain catalytic activity, as metal species can undergo deactivation through processes such as sintering or poisoning (Zhao, 2010). However, metal ions introduced via ion exchange are typically held on the zeolite structure through strong electrostatic interactions with the negatively charged framework. This stabilization might minimize the migration and agglomeration of metal species during thermal treatment, thereby reducing the likelihood of sintering (Urquieta-González et al., 2002; Li et al., 2022). Furthermore, the extra framework is accessible to the substrate and the products are not highly confined and are able to undergo desorption after the reaction.

Chen et al. (2024) conducted a study on the effects of manganese and zinc promoters on ferrite catalysts for CO_2_ hydrogenation. The reaction was carried out at 300°C, with a gas mixture ratio of CO_2_:H_2_ = 1:3 at a flow rate of 10 mL min^−1^ and a total pressure of 6 bar. They found that Mn additives led to a CO_2_ conversion of 11% and a selectivity towards C_2+_ hydrocarbons of 5%. (Yuan et al., 2023a). attempted to boost the production of light Olefins from CO_2_ Hydrogenation over Fe−Co bimetallic catalysts. They did this under the following conditions: 320°C, p = 3 MPa, gas hourly space velocity (GHSV) = 7,200 mL·gcat−1 h−1, and H_2_/CO_2_/N_2_ = 63/21. Their best catalyst FeCo-9:1 exhibited a selectivity of C_2+_ olefins over 36% and a CO_2_ conversion higher than 40%. In this study we investigated the effect of Co, Fe, and Mn on the activity of ZSM-5 in CO_2_ hydrogenation. The CO₂ hydrogenation process was carried out at temperature of 350°C and a pressure of 20 bar, introducing a high-pressure CO_2_/H_2_ gas mixture, in a 1:4 ratio, at a flow rate of 20 mL/min. The catalyst with the best activity was K-Mn/4Co-Fe-ZSM-5 with a CO_2_ conversion of 13% and 97% selectivity to C_2_H_4_.

## 4 Conclusion

In this investigation, Co, Mn, and K were successfully introduced into the extra-framework of Fe-based ZSM-5 zeolite. Various analytical techniques were employed to examine structural and morphological changes, including FTIR, pyridine FTIR, XRD, SEM, TEM, EDS, and XRF. The FTIR studies indicated a shift towards shorter wavelengths when metals were added, confirming the presence of Co, Fe, Mn, and K species. Pyridine FTIR revealed altered acidic sites, with a peak splitting indicating increased Lewis acidity post-metal modification. XRD diffractograms affirmed the retention of zeolite crystallinity, with characteristic MFI structures observed. BET analysis showed microporous characteristics, while the SEM and TEM images illustrated spherical shapes and confirmed uniform metal distribution. The catalytic performance results demonstrate that metal modifications significantly enhance both CO₂ conversion and olefin (C_2_H_4_) selectivity, with potassium-modified Mn/4Co-Fe-ZSM-5 achieving the highest conversion (13.2%) and selectivity (97.3%). The improved activity is attributed to the synergistic effects of metal species on active site formation and reaction pathways, particularly through promoting olefin production and minimizing methane formation. These findings underscore the potential of tailored metal modifications to optimize ZSM-5 catalysts for sustainable olefin production, offering a competitive edge in CO_2_ Hydrogenation techniques. The results show that Mn and K improved the selectivity of olefins by reducing the selectivity to CO on the Co-Fe-ZSM-5 catalyst.

## Data Availability

The original contributions presented in the study are included in the article/Supplementary Material, further inquiries can be directed to the corresponding author.
