# Supplementary material for: Olefin selectivity of K-Mn promoters on CoFe-ZSM-5 based catalyst in CO2 hydrogenation
Source: Front Chem. 2025 Feb 25;13:1562436. doi: 10.3389/fchem.2025.1562436 (PMC11893857; doi:10.3389/fchem.2025.1562436)
Supplement: Supplementary file 1 [file DataSheet1.docx]

Supplementary Material

# Supplementary Data

Structural and textural properties of catalysts.

*Figure S1: N_2_ adsorption/desorption isotherm of ZSM-5 catalysts*

*Figure S2: N_2_ adsorption/desorption isotherms of Fe-ZSM-5 catalysts*

*Figure S3: N_2_ adsorption/desorption isotherms of Co-Fe-ZSM-5 catalysts*

*Figure S4: N_2_ adsorption/desorption isotherms of Mn/Co-Fe-ZSM-5 catalysts*

*Figure S5: N_2_ adsorption/desorption isotherms of K-Mn/Co-Fe-ZSM-5 catalysts.*

*Figure S6: Pore size distribution of the ZSM-5 and modified ZSM-5 catalysts derived from desorption isotherm branch.*

Morphology and elemental characterization.

**
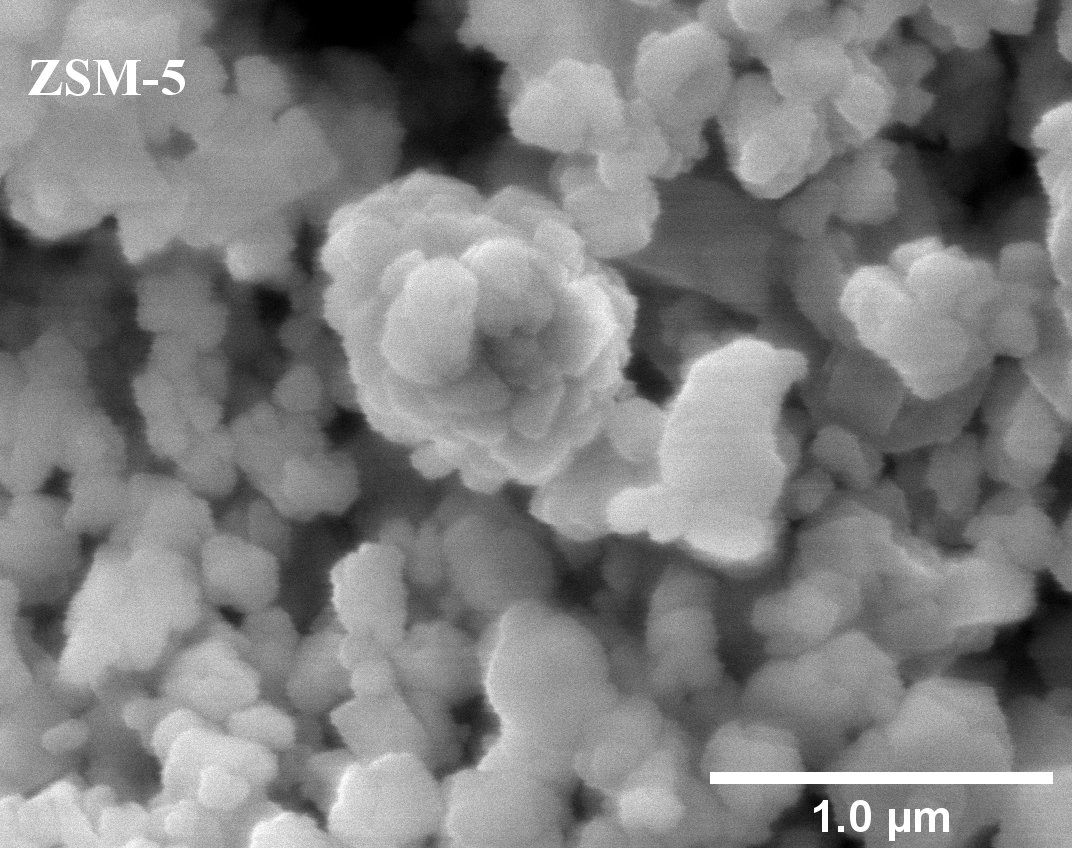
**

*Figure S7: SEM image of ZSM-5.*

**
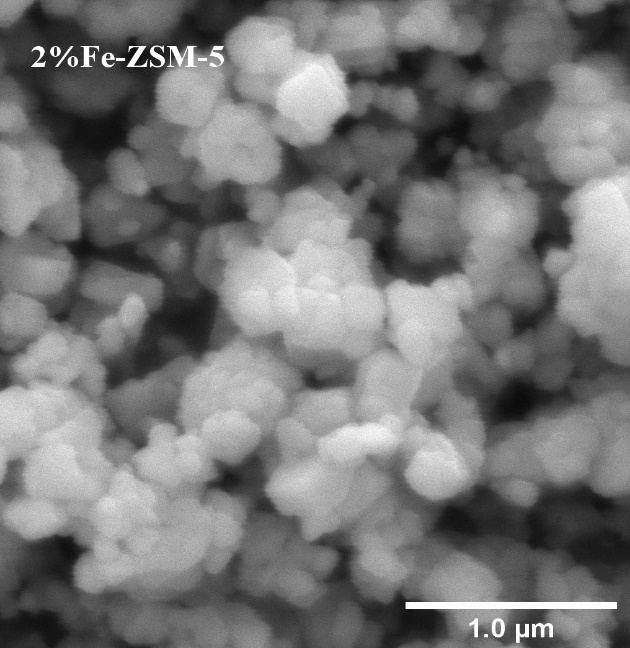

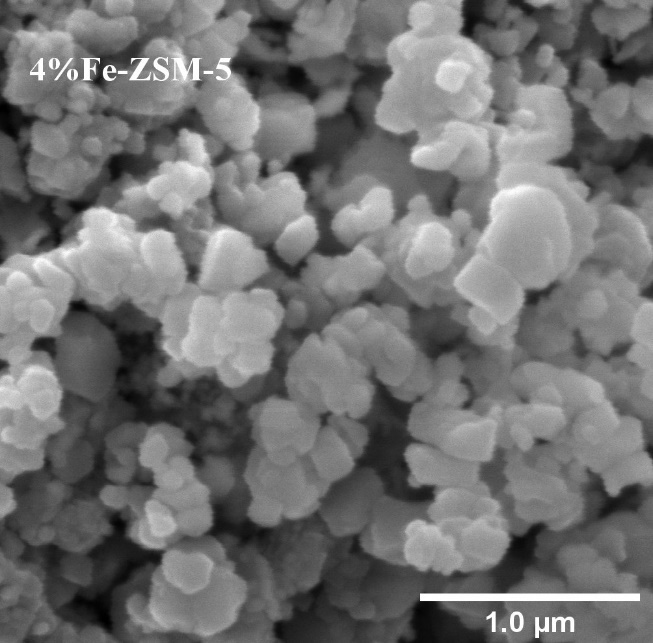
**

*Figure S8: SEM images Fe-ZSM-5.*

**
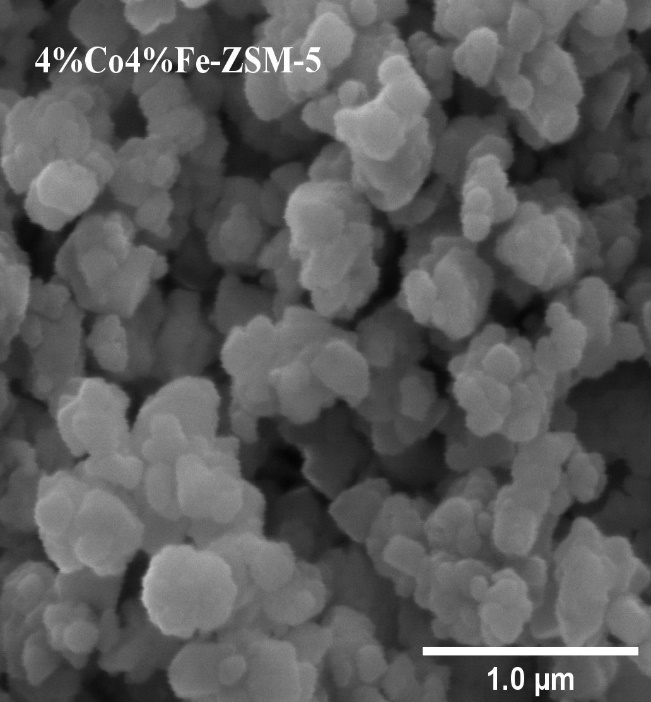
**

*Figure S9: SEM images of Co-Fe-ZSM-5.*

**
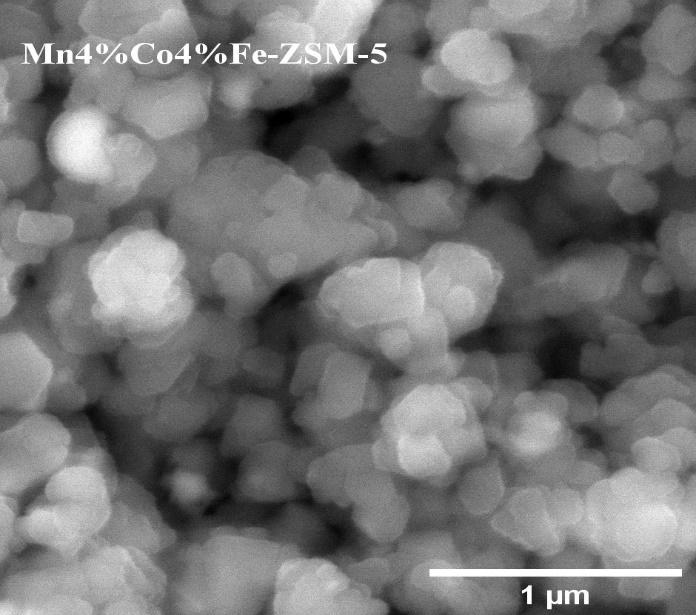
**

*Figure S10: SEM images of Mn-Co-Fe-ZSM-5.*

**
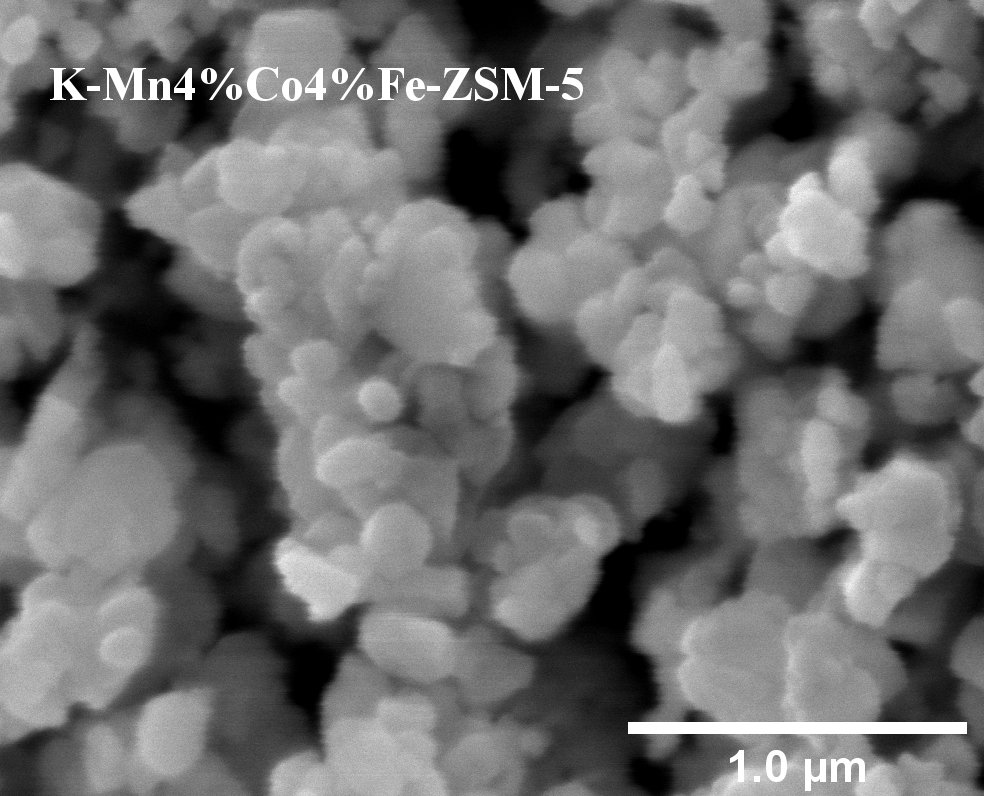
**

*Figure S11: SEM images of K-Mn-Co-Fe-ZSM-5.*


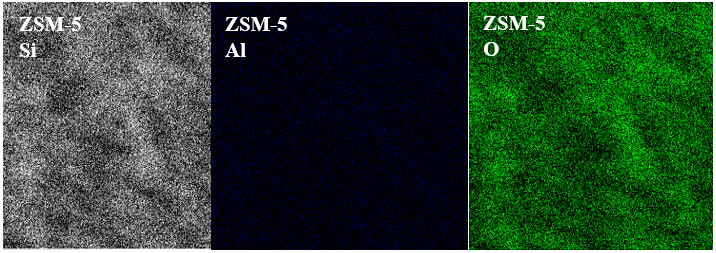


*Figure S12: EDX mapping images of ZSM-5.*


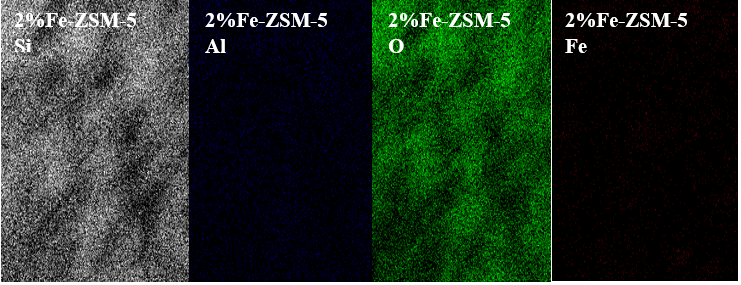


*Figure S13: EDX mapping images of Si, Al, O, and Fe for ZSM-5 and 2%Fe-ZSM-5, respectively.*


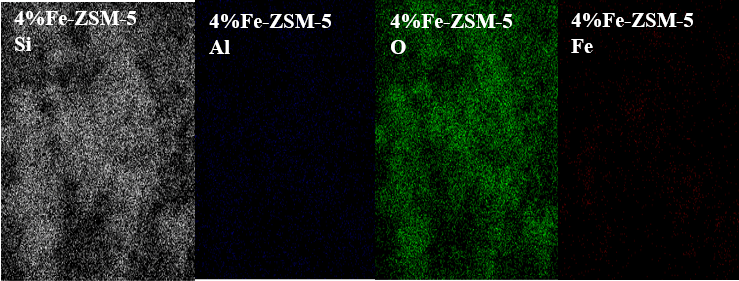


*Figure S14: EDX mapping images of Si, Al, O, and Fe for 4%Fe-ZSM-5.*


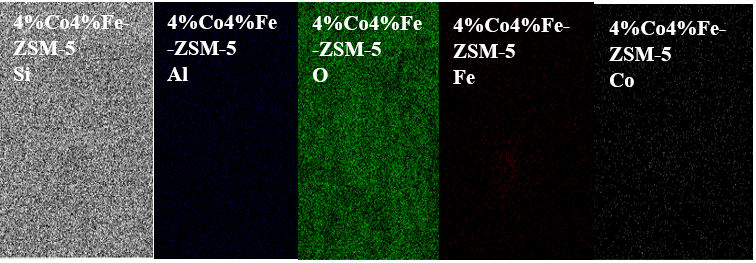


*Figure S15: EDX mapping images of Si, Al, O, Fe, and Co for 4%Co4%Fe-ZSM-5 .*


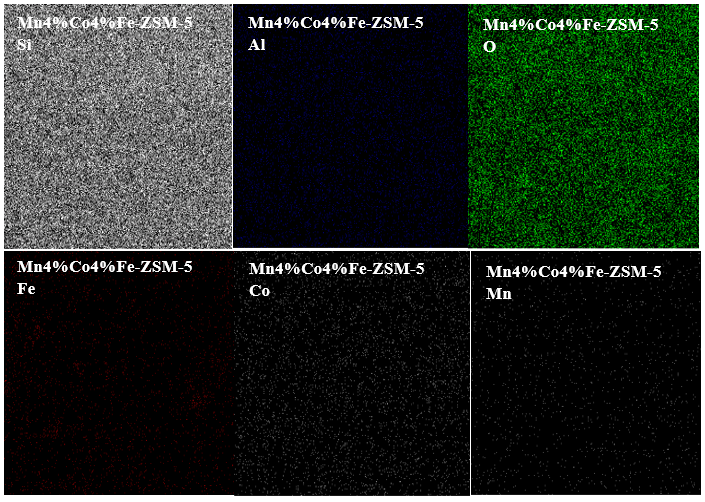


*Figure S16: EDX mapping images of Si, Al, O, Fe, Mn and Co for Mn4%Co4%Fe-ZSM-5 .*


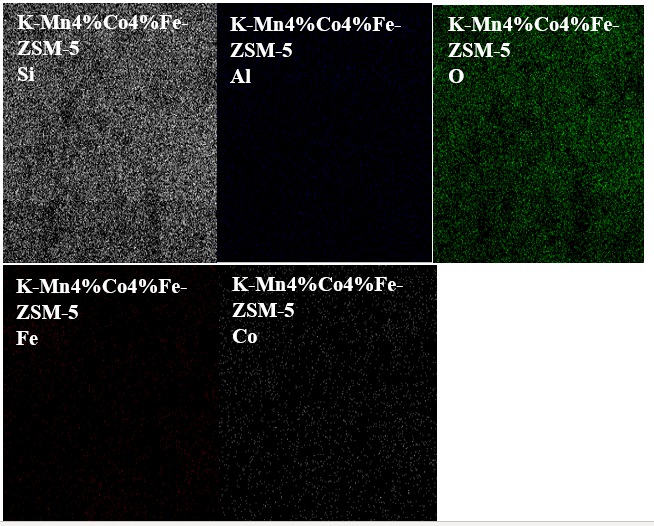


*Figure S17: EDX mapping images of Si, Al, O, Fe, and Co for K-Mn4%Co4%Fe-ZSM-5*


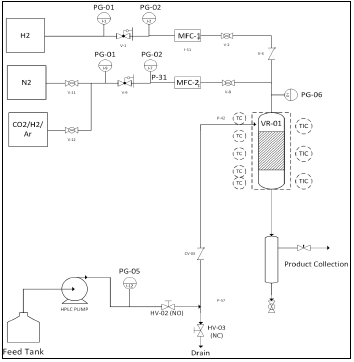


*Figure S18: Schematic experimental setups of the packed-bed flow reactor system for CO_2_ Hydrogenation.*


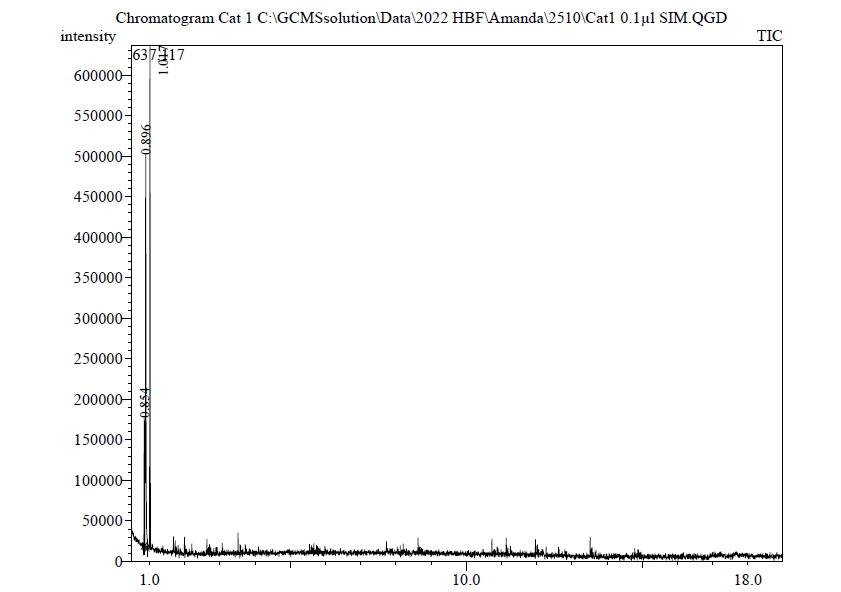


*Figure S19: GC Chromatogram from samples obtained from reactor studies with K-Mn/4Co4Fe-ZSM-5.*

***Table S1: Retention time and identified compounds on figure S2***

| Retention time (min) | Compound |
| --- | --- |
| 0.854 | Methanol |
| 0.896 | Ethene |
| 1.017 | Pentene |


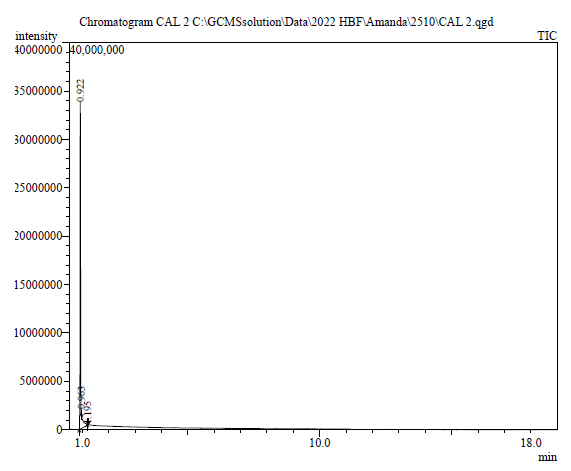


Pentene

*Figure S20: GC Chromatogram from samples obtained from reactor studies with Mn/4Co4Fe-ZSM-5.*


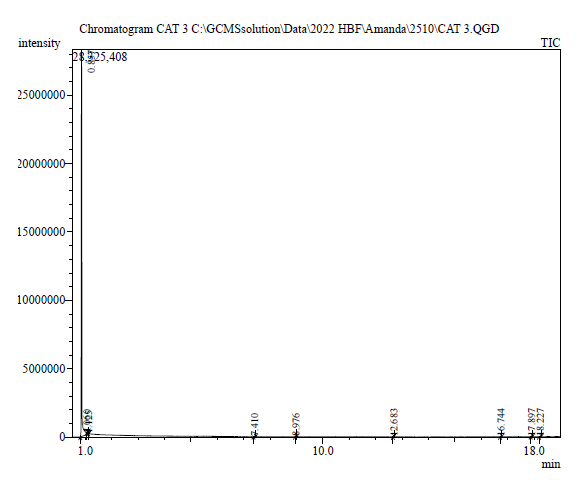


*Figure S21: GC Chromatogram from samples obtained from reactor studies with 2Co2Fe-ZSM-5.*

JCPDS files for PXRD: <https://drive.google.com/drive/folders/1EySQT0D7JMgbmynKGP3asalZWNL296i9?usp=drive_link>
